# Normative data and psychometric properties of the Patient Health Questionnaire-9 in a nationally representative Korean population

**DOI:** 10.1186/s12888-020-02613-0

**Published:** 2020-04-30

**Authors:** Cheolmin Shin, Young-Hoon Ko, Hyonggin An, Ho-Kyoung Yoon, Changsu Han

**Affiliations:** 1 Department of Psychiatry, Korea University College of Medicine, Korea University Ansan Hospital, 123, Jeokgeum-ro, Danwon-gu, Ansan-si, Gyeonggi-do 15355 Republic of Korea; 2 Department of Biostatistics, Korea University College of Medicine, Seoul, Republic of Korea

**Keywords:** PHQ-9, Depression, Standardization, Normative data, Nationally representative population

## Abstract

**Background:**

The Patient Health Questionnaire-9 (PHQ-9) has been standardized in several populations and is widely used in clinical practice and health care. However, it has not been appropriately standardized in the Korean general population, and no normative data have been presented. The aim of this study was to provide the normative data and psychometric properties of the PHQ-9 in the nationally representative population of Korea.

**Methods:**

We used the nationwide cross-sectional survey data of Korea from 2014 to 2016. The data of 10,759 individuals aged over 19 years were analyzed in this study. As the distribution of the PHQ-9 scores was not normative, the percentile ranks for raw scores were provided. The survey questionnaires included the PHQ-9, The EuroQol-5 Dimension (EQ-5D), and demographic characteristics. We analyzed the construct validity and internal consistency of the PHQ-9.

**Results:**

The normative data of the PHQ-9 were generated according to the sex and different age categories. The correlation coefficient between the sum of the PHQ-9 scores and the EQ-5D index was 0.44, which was moderate. The most appropriate model was the two-factor model with five ‘affective-somatic’ labeled items and four ‘cognitive’ labeled items. Cronbach’s α for the PHQ-9 was 0.79.

**Conclusions:**

Our result supports reliability and validity with two-factor structure of PHQ-9 for measuring depression in the Korean nationally representative population. The Korean normative data on the PHQ-9 according to percentile rank can assist in interpreting and comparing scores with other populations.

## Background

Depression is one of the most common mental health disorders [1]. It causes clinical morbidity in affected individuals and has serious consequences through increased mortality resulting from chronic illness and suicidal behavior [2, 3]. Depression also results in an economic burden due to functional impairment of patients and increased medical expenditure. Therefore, according to the World Health Organization, depression ranks second with respect to the global disease burden [1, 4]. Adequate evaluation of depression and provision of national and pan-social solutions for managing the disorder are crucial for promoting public mental health. In general, a clinician administered scale should be used in drug trials or practice settings to evaluate depression [5], but this would be costly and time-consuming. Therefore, self-report questionnaires with reasonable cost-effectiveness have been preferred for screening depression [6]. Therefore, various countries have made efforts to screen depression in the general population using simple and accurate instruments [7–9].

The Patient Health Questionnaire-9 (PHQ-9) is a multi-purpose, self-reporting instrument for screening and assessing depression. It consists of nine items based on the diagnostic criteria of major depressive episodes from the Diagnostic and Statistical Manual of Mental Disorders, 4th Edition [10]. Proper diagnosis of depression should be conducted using structured diagnostic interviews, such as the Mini-International Neuropsychiatric Interview or the Structured Clinical Interview for DSM-5 [11, 12], but screening instruments are necessary considering the time and cost involved, including that in training clinicians. Although the PHQ-9 can be used as a tool for diagnosis after obtaining the cut-off score for depression, it is widely used as a screening instrument as it can be self-administered [13].

The PHQ-9 was initially developed for primary care patients [10]; however, it has since proven to be a valid tool for the general population [14–17]. It is now widely used for screening depression in the general population and in the primary care setting.

Although the validity of the PHQ-9 has been demonstrated in certain Korean populations, including patients in primary care settings, patients with migraines, and in elderly patients [18, 19], the PHQ-9 has not been standardized for the general population. Furthermore, the psychometric properties of the PHQ-9 in the general population have not yet been provided. As the PHQ-9 is currently used to investigate depression in the nationwide survey of a national representative population, it is important to standardize the PHQ-9 and report its psychometric properties in the general population.

To interpret the results of the PHQ-9, an empirical PHQ-9 frame of reference for depressive symptoms is required. In other words, it should be possible to indicate through which norm the position of the individual who performed PHQ-9 in a specific population. In this case, data from the PHQ-9 can be used as easy-to-understand, basic data for interpreting results or for consultation with patients in the health care field. However, no normative data have been provided for the PHQ-9 in the general population of South Korea (hereafter referred to as “Korea”).

Normative data can be presented as standard scores (z or T scores); however, this may be inappropriate as psychological measures often do not have a normal distribution [20]. Data of depressive symptoms measured using instruments also have a positive skewness as most non-clinical populations report few symptoms [21]. According to a recent study, the PHQ-9 showed exponential distribution, as confirmed in studies conducted in the general population [22]. Thus, providing normative data for the PHQ-9 based on z or T scores would not be accurate. Hence, accurately determining the rank of an individual’s score in the population would be easy using a percentile rank.

This study aimed 1) to provide normative data for the PHQ-9 in a nationally representative Korean population by providing percentile ranks, based on the assumption that the PHQ-9 scores would not be normally distributed and 2) to examine the psychometric properties of the PHQ-9 as applied to the general population of Korea.

## Methods

We followed the “Strengthening the Reporting of Observational Studies in Epidemiology” (STROBE) guidelines for preparing this manuscript [23].

### Study population

The Korea National Health and Nutrition Examination Survey (KNHANES) is a cross-sectional, nationwide, population-based survey that monitors the health and nutritional status of the non-institutionalized population of Korea. The KNHANES uses a health interview, physical/laboratory examinations, and a nutrition survey. This health interview questionnaire gathers information on education, occupation, medical conditions, healthcare utilization, injuries, and quality of life, using a face-to-face interview method. It includes the use of self-reporting tools such as the PHQ-9 and the EuroQol-5 dimension (EQ-5D). In a specific time sequence, phases I (1998), II (2001), III (2005), IV (2007–2009), V (2010–2012), VI (2013–2015), and VII (2016–2018) of the surveys were conducted by the Korea Centers for Disease Control and Prevention of the Korean Ministry of Health and Welfare. A stratified multistage probability sampling design was used, and selections were made from sampling units based on geographical areas, sex, and age groups using household registries. The detailed survey protocol has been previously described [24].

This study was based on the data from the sixth and seventh KNHANES, which used the PHQ-9 as a screening instrument for depression. The sixth and seventh KNHANES administered the PHQ-9 to adults aged 19 years and over in 2014 and 2016, respectively. This study was approved by the Korea University Institutional Review Board, and all participants provided written informed consent before their enrollment in the survey.

### Study instruments

#### Measurement of depressive symptom (PHQ-9)

The PHQ-9 comprises nine items and is used to screen, monitor, and measure the severity of depression. Each item has 4-point response options that are checked as “0 = not at all,” “1 = several days,” “2 = more than half days,” and “3 = nearly every day” depending on the level on concern due to depressive symptoms in the last 2 weeks. The sum of the scores could range from 0 to 27. In addition, the validation of the PHQ-9 as a screening tool for the general population was conducted in several separate studies [14, 15, 17]. A PHQ-9 score of 10 or more had an 88% sensitivity and an 88% specificity to detect major depression in a general population including people of various ethnicities [10]. Furthermore, a meta-analysis reported that a cut-off point of 10 or more had a sensitivity of 80–90% and was generally considered to indicate a detecting major depression [25]. In addition, Kroenke et al. (2011) suggested that mild, moderate, moderately severe, and severe depression were represented by the PHQ-9 scores of 5, 10, 15, and 20, respectively. Therefore, we also presented the prevalence according to the severity of depression based on these scores.

#### EQ-5D

The EQ-5D is a short, self-rating questionnaire used to subjectively describe and evaluate the health-related quality of life; it is generally used as an outcome measure in both clinical and health care service research [26]. The EQ-5D provides a descriptive profile of the health-related quality of life and a subjective overall rating of the patient’s own health status on the day of administration using a visual analog scale. In the sixth and seventh KNHANES, only an ED-5D descriptive system, which consists of five items that measure five dimensions of health including mobility, self-care, usual activities, pain/discomfort, and anxiety/depression, was administered. Each dimension is represented by an item with the following three response levels: no problem, some problems, and extreme problems. According to a specific set of preference values based on surveys in the general population, a single index score (EQ-5D index) is assigned to all possible descriptive profiles of the EQ-5D. A previous study reported the EQ-5D index, which reflected the preferences of a representative Korean population for the EQ-5D health states [27]. The Korean version of the EQ-5D has been developed, and its validity and reliability have been proven in patients with several clinical populations [28, 29].

### Statistical analysis

To characterize the representative population of Korea, the sampling weights assigned to the subjects were applied to all analyses and were generated by considering the complex sample design, non-response rate of the target population, and post-stratification. In previous studies on the PHQ-9 [15, 30], if the missing value was less than 20%, the missing value was replaced with the average of the remaining items. If the number of items missing from the scale exceeded 20% of the total number of items, they were not counted in the total score and were treated as missing data.

For descriptive statistics, means, standard deviations, and frequencies were calculated for sociodemographic factors. To investigate the differences between groups according to sociodemographic characteristics, the χ^2^-test and Kruskal-Wallis-test were performed. The normality distribution according to variables was tested using the Kolmogorov-Smirnov test. The effect size of sociodemographic factors with significant differences was interpreted according to Cohen [31]. The subgroups for each variable, with the highest and lowest mean scores, were considered to calculate the value of Cohen’s d, which represents the difference between the means divided by the standard deviation.

For reliability, the internal consistency of the PHQ-9 was assessed. To determine the construct validity, we analyzed the correlation between the PHQ-9 and EQ-5D. To investigate the factor structure of the nine PHQ-9 items, total sample was randomly partitioned into 2 subsamples, each 5379 and 5380 subjects. Exploratory factor analysis (EFA) using maximum likelihood estimation was applied in the first subsample to examine which factor structure is generated, because this is the first study to investigate the PHQ-9 in the nationally representative population of Korea. Oblique rotation was conducted due to possibility of correlation between factors. The sample’s adequacy was assessed by the Kaiser–Meyer–Olkin (KMO) measure of sampling adequacy. Based on the result of EFA, confirmatory factorial analysis (CFA) was conducted to present the criteria, including the root mean square error of approximation (RMSEA) [32], the comparative fit index (CFI) [33], and the Tucker-Lewis index (TLI) [34].

To provide normative data for the PHQ-9 as percentile ranks, percentile numbers with respect to age and sex were generated for the total score. To investigate the distribution of depression severity, the total sample was categorized, as recommended by Kronke et al. [10]. The PHQ-9 score was categorized into scores of 0–4, 5–9, 10–14, 15–19, and 20 or greater, which indicated “minimal,” “mild,” “moderate,” “moderately severe,” and “severe” depressive symptoms, respectively.

The statistical analysis, excluding CFA, was performed using SPSS for Windows, version 20.0 (IBM Corp, Armonk, NY, USA). CFA was conducted using R 3.5.1 software (The R Foundation for Statistical Computing). A *p*-value < 0.05 was considered significant.

## Results

### Study population characteristics

**Fig. 1:**
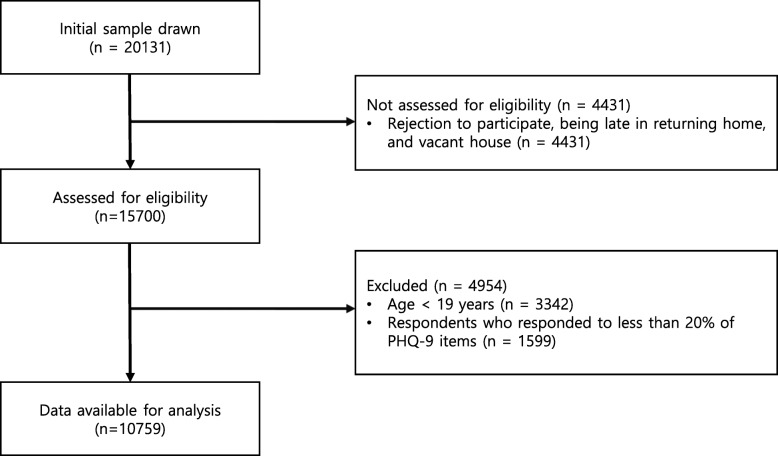
Flow diagram of the inclusion of participants based on the STROBE guidelines

Of the 15,700 people who participated in the KNHANES from 2014 to 2016, 12,358 were aged over 19 years. Among them, responders who failed to respond to more than 20% of the PHQ-9 items (*N* = 1599) were excluded (Fig. 1). Data of 10,759 people were used for the final analysis. Table 1 illustrates the association of PHQ-9 scores with sociodemographic characteristics. Higher PHQ-9 scores were significantly associated with sex, age, years of education, employment status, household income, marital status, and cohabitation in the general population. The calculated effect sizes were low for sex, age, years of education, employment status, household income, and cohabitation and were moderate to high for marital status.

**Table 1:** Sociodemographic characteristics of the study sample and its association with PHQ-9 scores

	N (%)	PHQ-9 mean (SEM)	Group difference	Cohen’s effect size
Sex			*P* < 0.001*	0.29
Male	4543 (49.1)	2.14 (0.05)		
Female	6216 (50.9)	3.28 (0.05)		
Age group			*P* < 0.001*	0.24
19–29	1249 (18.5)	3.09 (0.08)		
30–39	1893 (18.8)	2.96 (0.06)		
40–49	1933 (20.6)	2.31 (0.07)		
50–59	2009 (19.9)	2.45 (0.08)		
60–69	1857 (12.0)	2.66 (0.08)		
70–79	1439 (7.9)	3.21 (0.1)		
80+	379 (2.2)	3.37 (0.21)		
Education years			*P* < 0.001*	0.29
6	2441 (15.8)	3.57 (0.1)		
9	1135 (9.2)	2.85 (0.13)		
12	3483 (37.2)	2.66 (0.05)		
18	3689 (37.8)	2.45 (0.06)		
Employment			*P* < 0.001*	0.24
Yes	6319 (62.9)	2.39 (0.04)		
No	4434 (37.1)	3.34 (0.07)		
Household income			*P* < 0.001*	0.24
Lowest	2051 (15.3)	4.18 (0.12)		
Second	2664 (23.9)	2.86 (0.06)		
Third	3015 (30.2)	2.39 (0.05)		
Highest fourth	2999 (30.6)	2.28 (0.05)		
Habitation area			*P* = 0.633	0.02
Urban	8711 (84.3)	2.73 (0.04)		
Rural	2048 (15.7)	2.81 (0.12)		
Marital status			*P* < 0.001*	0.68
Married	7677 (67.1)	2.39 (0.04)		
Separated	54 (0.5)	4.78 (0.74)		
Widowed	953 (6.0)	3.83 (0.15)		
Divorced	419 (3.7)	4.60 (0.22)		
Never married	1652 (22.7)	3.16 (0.09)		
Cohabitation			*P* < 0.001*	0.38
None	1128 (8.7)	4.01 (0.12)		
1	3179 (24.2)	2.79 (0.07)		
2 or more	6452 (67.1)	2.56 (0.04)		

Values are presented as unweighted frequency, weighted percentage, and weighted mean (SEM). * indicates *p* < 0.001

### Normative data displayed by percentile ranks for the PHQ-9 total score

**Fig. 2:**
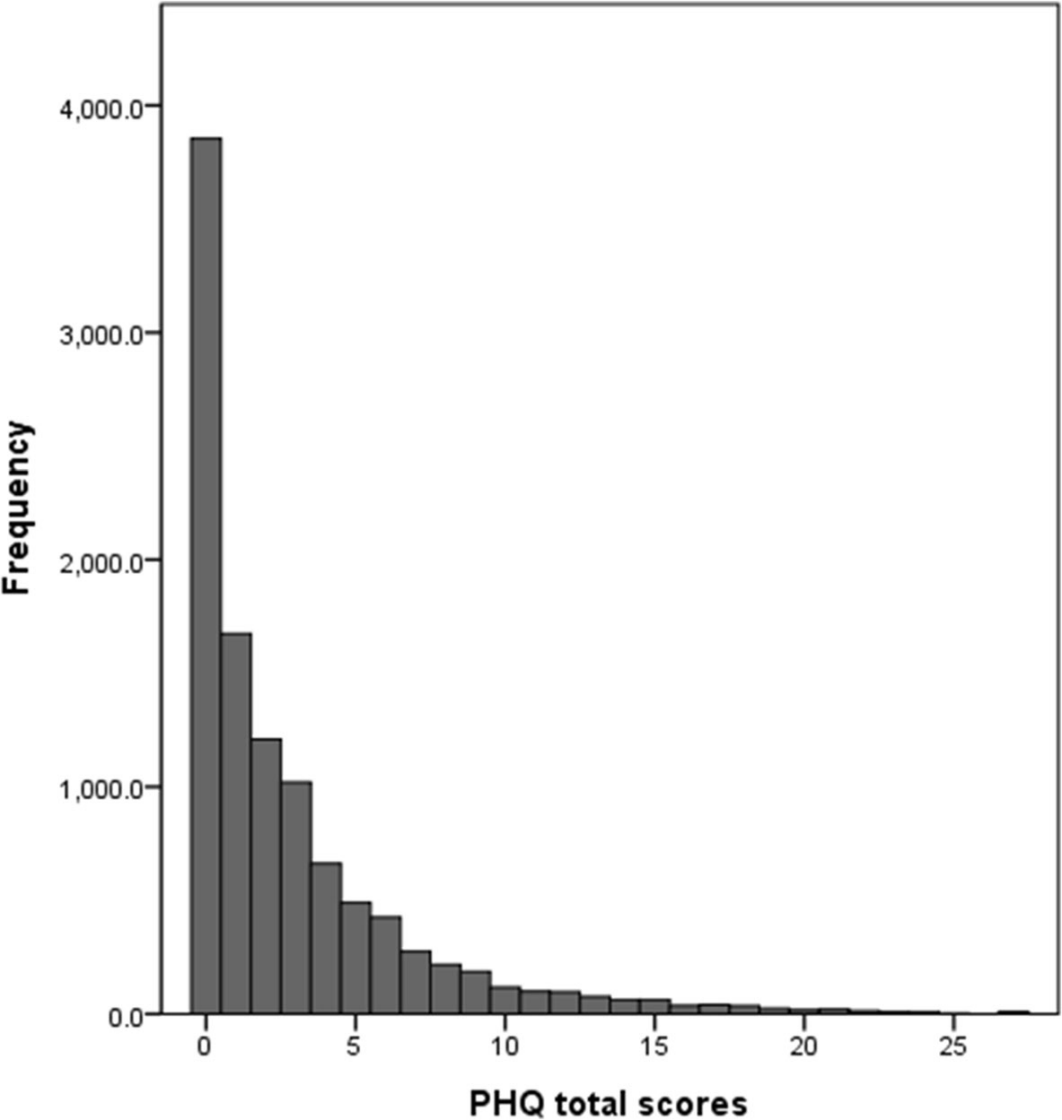
Distribution of the total PHQ-9 scores in a nationally representative Korean population (*N* = 10,759)

The distribution of the PHQ-9 total score was strongly left-skewed, congregating at the 0 point (Fig. 2). Table 2 presents the normative data with respect to age group and sex. The presented percentile ranks indicate an individual’s PHQ-9 score in the general population with respect to sex and age.

**Table 2:** Normative data of the PHQ-9 in the general Korean population

	Total	Men								Women							
	*n* = 10,759	19–29 *n* = 522	30–39 *n* = 792	40–49 *n* = 827	50–59 *n* = 812	60–69 *n* = 815	70–79 *n* = 630	80- *n* = 145	Total *n* = 4543	19–29 *n* = 727	30–39 *n* = 1101	40–49 *n* = 1106	50–59 *n* = 1197	60–69 *n* = 1042	70–79 *n* = 809	80- *n* = 234	Total *n* = 6216
M	2.74	2.37	2.57	1.98	1.96	1.90	2.11	2.40	2.17	3.88	3.37	2.64	2.92	3.35	4.02	3.87	3.29
SEM.	0.04	0.1	0.09	0.1	0.09	0.11	0.09	0.28	0.05	0.14	0.09	0.09	0.1	0.11	0.16	0.26	0.05
Sum Score	Percentile																
0	35.3	35.2	34.4	41.8	45.4	51.8	49.2	45.6	41.5	20.3	23.7	33.2	31.2	34.7	36.1	34.5	29.3
1	51.4	53.3	50.9	60.0	61.9	65.2	63.1	56.4	58.0	33.8	39.4	50.8	51.3.	48.0	46.7	45.6	45.0
2	63.1	68.8	64.2	73.9	72.7	72.9	71.3	64.7	70.0	46.7	51.6	64.9	62.0	59.4	52.4	50.7	56.5
3	72.8	78.6	74.4	82.3	81.5	83.0	81.0	77.5	79.8	57.3	61.8	73.6	71.2	68.1	61.9	64.0	66.1
4	78.8	82.0	80.7	85.8	86.5	86.3	85.5	82.2	84.2	66.2	73.2	80.1	77.7	73.3	68.3	67.7	73.7
5	83.9	86.7	86.9	89.7	90.6	90.9	87.7	84.9	88.7	74.7	79.7	85.5	82.1	77.7	71.6	71.1	79.2
6	87.8	90.6	89.2	92.0	92.5	93.1	91.2	89.0	91.3	80.8	85.0	89.0	87.5	82.3	77.8	78.8	84.4
7	90.3	93.0	91.5	94.0	93.7	94.5	93.0	92.1	93.2	84.9	89.4	91.5	89.4	84.7	80.1	83.4	87.5
8	92.3	94.7	92.7	96.1	95.4	95.6	94.7	94.1	94.8	87.2	91.7	94.3	91.2	87.4	82.7	85.1	89.8
9	93.8	95.7	94.5	96.9	96.2	96.2	95.7	95.4	95.9	89.5	93.3	95.7	93.3	90.1	85.4	87.9	91.9
10	95.0	96.8	95.9	97.6	96.7	96.7	96.3	95.8	96.7	92.5	94.3	96.0	94.5	92.2	87.7	88.9	93.3
11	95.9	97.7	96.4	98.3	97.5	96.9	96.8	96.4	97.3	94.1	96.0	96.5	95.1	93.0	89.6	90.9	94.5
12	96.7	98.0	97.0	98.7	98.2	97.3	97.6	95.8	97.9	95.4	97.1	97.3	95.7	93.9	91.7	92.6	95.5
13	97.4	98.4	98.5	99.0	98.7	97.9	98.0	96.4	98.5	96.7	97.4	97.6	96.6	95.2	93.0	93.6	96.4
14	97.9	99.1	99.0	99.2	99.4	98.0	98.0	98.0	99.0	97.0	97.8	98.0	97.1	96.3	93.8	94.4	96.9
15	98.4	99.3	99.2	99.2	99.6	98.7	98.2	99.4	99.2	98.4	98.1	98.4	97.9	97.1	95.0	95.9	97.7
16	98.7	99.5	99.3	99.4	99.6	98.9	98.5	99.4	99.3	98.9	98.3	98.5	98.2	97.6	96.0	96.1	98.1
17	99.0	99.5	99.6	99.8	99.6	99.2	98.5	99.4	99.5	99.1	98.5	98.9	98.9	98.1	97.1	97.6	98.6
18	99.2	99.6	99.6	99.8	99.6	99.4	98.6	99.4	99.6	99.4	98.9	98.9	99.0	98.7	98.0	97.9	98.9
19	99.4	99.6	99.6	99.8	99.6	99.6	98.8	100.0	99.6	99.7	99.3	99.1	99.3	99.1	98.7	98.3	99.2
20	99.5	99.7	99.8	99.8	99.6	99.6	99.0	100.0	99.7	99.7	99.5	99.3	99.6	99.4	98.8	98.9	99.4
21	99.7	99.7	100.0	99.8	99.6	99.6	99.2	100.0	99.7	100.0	99.5	99.6	99.7	99.7	99.1	99.6	99.7
22	99.8	99.7	100.0	99.8	99.9	99.9	99.9	100.0	99.9	100.0	99.7	99.6	99.9	99.7	99.1	99.6	99.7
23	99.8	100.0	100.0	100.0	99.9	99.9	99.9	100.0	99.9	100.0	99.9	99.7	99.9	99.7	99.2	100.0	99.8
24	99.9	100.0	100.0	100.0	100.0	99.9	100.0	100.0	100.0	100.0	99.9	99.9	99.9	99.8	99.5	100.0	99.9
25	99.9	100.0	100.0	100.0	100.0	99.9	100.0	100.0	100.0	100.0	99.9	100.0	100.0	99.8	99.5	100.0	99.9
26	99.9	100.0	100.0	100.0	100.0	99.9	100.0	100.0	100.0	100.0	99.9	100.0	100.0	99.8	99.5	100.0	99.9
27	100.0	100.0	100.0	100.0	100.0	100.0	100.0	100.0	100.0	100.0	100.0	100.0	100.0	100.0	100.0	100.0	100.0

Values are presented as unweighted frequency, weighted percentile rank, and weighted mean (SEM)

### Internal consistency and construct validity

The internal consistency parameter (Cronbach’s α) of the PHQ-9 was calculated to be α = 0.79. The correlation between the PHQ-9 total score and the EQ-5D score is presented in Table 3. Depression assessed using the PHQ-9 showed the highest correlation with the mental component (depression and anxiety) of the EQ-5D (*r* = 0.475, *p* < 0.001). The correlation between the PHQ-9 total score and the EQ-5D index was 0.428 (*p* < 0.001).

**Table 3:** Correlations of depression and health-related quality of life (*N* = 10,759)

	Depression (Sum of the PHQ-9 scores)
Mobility	0.265**
Self-care	0.206**
Usual activities	0.296**
Pain/discomfort	0.336**
Anxiety/depression	0.475**
EQ-5D index	0.428**

** *P* < 0.001

### Factor analysis

The results of EFA displayed a two-factor structure. The eigenvalue of the two factors was over 1.0 (3.8 and 3.2). The KMO measure of sampling adequacy was 0.88. Bartlett’s test of sphericity showed a χ2 of 12,768.33 (*p* < 0.001). Overall, this model explained a 53.5% variance. The variance accounting for the other seven factors was less than 53.5%, and it ranged from 4.7 to 9.1%. The scree test showed a sharp drop after identifying two factors. Table 4 presents the factor loadings for a two-factor model, developed by EFA.

**Table 4:** Standardized factor loadings for the two-factor model

Factor loadings	*β*
Affective–somatic	
Item 1. Anhedonia	0.61
Item 2. Depressed mood	0.57
Item 3. Sleep disturbance	0.70
Item 4. Fatigue	0.78
Item 5. Poor appetite/overeating	0.65
Cognitive	
Item 6. Feeling guilty	0.68
Item 7. Poor concentration	0.63
Item 8. Psychomotor agitation/retardation	0.73
Item 9. Thoughts of death	0.73

This result was tested for goodness of fit by CFA (fit statistics: χ^2^ = 710.97, df = 26, TLI = 0.908, CFI = 0.933, RMSEA = 0.070 [90% CI: 0.066–0.074]). The first factor represented affective-somatic symptoms (anhedonia, depressed mood, sleep disturbance, fatigue, poor appetite/overeating), and the second factor (feeling guilty, poor concentration, psychomotor retardation/psychomotor agitation, suicidal ideation) represented cognitive symptoms. The latent correlation between the factors was 0.66. As suggested by previous studies in the general population and primary care patients [15, 17], we also tested one-factor model. The fit of the unidimensional model was not as reasonable as the data (fit indices: χ^2^ == 1035.19, df = 27, TLI = 0.869, CFI = 0.902, RMSEA = 0.083 [9% CI: 0.079–0.088]) presented in the multidimensional model described above.

### Distribution of depressive symptoms measured using the PHQ-9

The percentage of individuals with no depressive symptoms (PHQ-9 score = 0) was 35.1%. The prevalence rates of depressive symptoms according to the recommended cut-off points for minimal (scores of 1–4), mild (scores of 5–9), moderate (scores of 10–14), moderately severe (scores of 15–19), and severe (scores of 20–27) symptoms were 43.5, 14.9, 4.4, 1.5, and 0.6%, respectively.

## Discussion

This is the first study to present the normative data of the Korean national representative population for the PHQ-9. Korea has a well-established national screening system [35], and the PHQ-9 is used as a screening tool for annual health examination programs and to detect depressive symptoms in national surveys [36]. Therefore, it is necessary to interpret the severity of the total PHQ-9 score obtained through a screening program. Through this study, it was possible to determine the specific percentile rank of the PHQ-9 score for a Korean individual. Percentile ranks could be used to examine the percentage standing of an individual with a particular score. This enables clinicians or researchers to easily interpret the abnormality of an individual’s score. For example, a 45-year-old man with an 8-point PHQ-9 score in our study data showed a 91.8 percentile rank among the whole population and 95.9 percentile rank among the men in his age group. It can also be used to compare PHQ-9 scores across different nationally representative samples. Two previous studies provided normative data for the PHQ-9 in a nationally representative population of Germany [15, 37]. Rief et al. presented the PHQ-9 score in percentile [37], while Kocalevent et al. presented the percentile rank for the PHQ-9, as presented in this study [15]. According to the data of a previous German study on a nationally representative sample [15], 45-year-old men with a PHQ-9 score of 8 showed a 94.1 percentile rank according to their sex and age group. Therefore, the number of Korean men in their 40s with a PHQ-9 score of 8 is lower than that of German men in the same age group. Thus, it was confirmed that the PHQ-9 score of an individual can be easily compared in the same demographic groups between countries.

We provided age-specific and sex-specific normative data because previous studies have indicated that depressive symptoms are differently distributed according to age [38, 39] and sex [40]. In our sample, mean scores of the PHQ-9 were greater in women than in men and were distributed in a U-shape according to age group. Likewise, the percentile ranks of certain points of PHQ-9 normative data were generally greater in women than in men. The percentile ranks were higher in the youngest and oldest age groups and lower in the middle age groups.

Normative data of the PHQ-9 are of importance in primary care setting. Identifying the standard of depressive symptoms in the community is a strong evidence-based approach in the management of these patients. Additionally, normative data can describe the natural history of clinical conditions in the community [41]. Further, screening of depression is widely recommended in the primary care setting [42]; thus, our results support that screening with the PHQ-9 in this setting is appropriate.

Factor analysis showed that the PHQ-9 represented two-factor structure in the general population of Korea. Each of the two-factor models fit significantly better than the one-factor model. Depressive symptoms evaluated using the PHQ-9 are best divided into affective-somatic symptoms and cognitive symptoms in the Korean general population. The PHQ-9 has been shown difference in the factor structure according to the study population. Unlike the present study, several previous studies have reported unidimensional structure of the PHQ-9 in the general population and primary care patients [16, 17]. Kocalevent et al. also supported that a one-factor model is valid for the PHQ-9 in a nationwide representative sample [15]. Previous studies have shown slightly different two-factor models that generally consisted of one factor representing somatic items (sleep disturbance, fatigue, and appetite change) and the other factor representing non-somatic items (anhedonia, depressed mood, poor concentration). Those findings were mostly derived from a clinical sample with somatic diseases [43–46]. However, this was not the case in our sample, even though it could be divided according to affective-somatic and cognitive symptoms. This is an interesting finding because heterogeneous samples such as the general population result in greater correlation between factors, and the single items in the PHQ-9 will tend to load on one factor. A similar factor structure was derived from a study that previously conducted a factor analysis of the Beck depression inventory for college students in Korea [47], and it is necessary to find out through subsequent studies whether this is due to the unique cultural background or the characteristics of the Korean population. Although the current study supported a two-factor model of the PHQ-9 in the nationally representative Korean population, using two factor scores may not be optimal in screening. The utility of using PHQ-9 total score in screening have been well developed and the PHQ-9 is widely used as screening tool [13, 42]. Also, given that the factors were moderately correlated in our data, it would complicate interpretation of corresponding test scores for screening.

We found that the PHQ-9 could be used for self-administered measurement of depression with good reliability and validity in a nationally representative Korean population. This study presented all possible psychometric properties to properly interpret the results obtained by using the PHQ-9. Our finding of good internal consistency is also consistent with those of previous studies on the general population [15, 17]. The correlation between the PHQ-9 and EQ-5D was 0.43, which is similar to the results of other studies that examined construct validity by assessing the correlation between the scales evaluating quality of life and the PHQ-9 [10, 14, 15]. The KNHANES sample used in this study is representative of the Korean population, and the validation data obtained through this study sample can be said to be the result of that reliable validation study. The PHQ-9 has been standardized across populations; however, few studies have standardized the PHQ-9 in a national representative sample. The results obtained from these studies may help facilitate comparisons in the general population across countries. To date, the PHQ-9 has been primarily standardized in a nationally representative sample of Germany. In addition, studies on the general populations of Germany, Hong Kong, and China have been conducted for standardization, and those studies showed that the PHQ-9 presented sound reliability and validity in the general population [14–17].

The prevalence of mild depressive symptoms assessed with the PHQ-9 was 14.9%, which was almost the same as that reported in the 2005–2008 national survey in the United States [48]; however, this prevalence was lower than that of mild depressive symptoms in Germany (18.1%) and higher than that in Hong Kong (13.7%). The prevalence for moderate to severe depressive symptoms was 6.2%. We previously reported a prevalence rate of 6.7% based on the 2014 KNHANES data [36], and it was slightly lowered with the pooled data from 2014 and 2016. It was similar or greater than those observed in the general populations of Germany (6.1%), Latvia (6.2%) and Hong Kong (4.3%) [17, 49, 50] and was lower than that in the general population of the United States (8.1%) [48, 51]. The prevalence of depression has gradually increased in Korea for decades, resulting in a prevalence similar to that in Western countries. According to nationwide epidemiological surveys conducted every 5 years, the life time prevalence of major depressive disorder was 4.0% in 2001 and increased to 6.7% in 2011. Constant modernization over the decades along with the rapid aging of society may be related to the increased prevalence of depression [52–54].

This study has its limitations. First, the characteristics of the sample was a limitation. Although the KNHANES reports on the results of household surveys, it does not include the data of populations in correctional facilities, hospitals, and nursing homes. Therefore, when comparing normative data with those of any other country, the procedure for sampling the general population in each country must be known. Second, we did not evaluate the validity of the PHQ-9 with the standard criterion of clinical interviews, which involves the calculation of the specificity and sensitivity for an optimal cut-off point and plotting of a receiver operating characteristics curve. Further, no cut-off point for depression has been determined in the Korean general population; thus, we were not able to calculate the prevalence of major depression. It should be noted that the range of points of depression severity (minimal, mild, moderate, moderately severe, and severe) used in this study had been suggested in a previous study [10]. There is scope for further studies to determine the cut-off points of depressive disorders by using standard criterion interviews in the general Korean population. Moreover, it was difficult to present the predictive validity of the PHQ-9 due to the lack of standard criterion interviews.

## Conclusions

The results of this study suggest that in a nationally representative population, normative data of percentile rank generated using the PHQ-9 are useful for interpreting the severity of depressive symptoms on the PHQ-9. Normative data can also be used to compare the severity of depressive symptoms with that in other countries or populations. Our results provide evidence on the psychometric properties of the PHQ-9 that supports its utility as a valid and reliable measurement for depression in the general population of Korea. It is expected that the PHQ-9 will be suitable for mass screening programs for depressive symptoms in the general population.

## Data Availability

All raw data from the survey are available at http://knhanes.cdc.go.kr/. The datasets used and/or analyzed during the current study are available from the leading author on reasonable request.

## Abbreviations

PHQ-9: Patient Health Questionnaire-9
KNHANES: Korea National Health and Nutrition Examination Survey
EQ-5D: EuroQol-5 Dimension
EFA: Exploratory Factor Analysis
CFA: Confirmatory Factor Analysis
RMSEA: Root Mean Square Error of Approximation
CFI: Comparative Fit Index
TLI: Tucker-Lewis Index
